# Off-Label Use of 0.19 mg Fluocinolone Acetonide Intravitreal Implant: A Systematic Review

**DOI:** 10.1155/2021/6678364

**Published:** 2021-05-17

**Authors:** Flávio Ribeiro, Manuel S. Falcão

**Affiliations:** ^1^Faculty of Medicine of the University of Porto, Porto, Portugal; ^2^Department of Surgery and Physiology, Faculty of Medicine, University of Porto, Porto, Portugal; ^3^Department of Ophthalmology, Centro Hospitalar Universitário São João, Alameda Professor Hernâni Monteiro, Porto 4200-319, Portugal

## Abstract

Corticosteroids are used in a variety of ophthalmological diseases. One challenge faced by ophthalmologists is to deliver corticosteroids to the posterior segment of the eye with efficacy and safety. Sustained-release corticosteroid implants may be the answer to this problem. The 0.19 mg fluocinolone acetonide (FAc) implant (Iluvien®) releases FAc for 36 months, and it is approved for the treatment of diabetic macular edema (DME) and noninfectious uveitis. We decided to do a systematic review to acknowledge in which other diseases FAc implant is being used off-label. A literature search was performed in the following three electronic databases: PubMed, Scopus, and Web of Science (from January 1^st^, 2000, to September 20^th^, 2020), using the following query: (“Fluocinolone Acetonide” OR Iluvien®) AND (“eye” OR “ocular” OR “intravitreal).” A total of 11 papers were included, and the use of FAc implant was analyzed in the following diseases: radiation-induced maculopathy (RM); paraneoplastic visual syndromes (melanoma-associated retinopathy (MAR) and cancer-associated retinopathy (CAR)); Sjogren's syndrome-related keratopathy; retinal vein occlusion (RVO); cystoid macular edema (CME); diabetic retinal neurodegeneration (DRN); and retinitis pigmentosa (RP). FAc implant may be a potential treatment for these diseases; however, the level of scientific evidence of the included studies in this review is limited. Further studies with larger cohorts and longer follow-ups are needed to validate this data.

## 1. Introduction

The anti-inflammatory, antiangiogenic, antiproliferative, antiedematous, and antiapoptotic properties of corticosteroids make them a useful drug class for a wide spectrum of eye diseases, such as diabetic macular edema (DME), cystoid macular edema (CME), retinal vein occlusion (RVO), and uveitis [1, 2]. With the increase in life expectancy, these diseases are becoming more prevalent, which creates a challenge for ophthalmologists due to their substantial impact on the quality of life of these patients [3, 4].

The other challenge faced by ophthalmologists is the administration of corticosteroids to the posterior segment of the eye [5]. The anatomy of the eye and its physiologic aspects make it difficult for drugs to be absorbed into the eye. Topical administration is useful in conditions affecting the anterior segment of the eye, but it does not meet the needs of the posterior segment [6]. One other alternative is the systemic route, but the inner retina and outer retina blood-retinal barriers are important obstacles for drug molecules [6]. High doses of systemic corticosteroids are needed to achieve therapeutic levels in the eye, but this is not always feasible due to the adverse effects of corticosteroids [7]. Intravitreal injections eliminate the risk of systemic adverse effects while allowing therapeutic drug levels in the vitreoretinal space. However, intraocular clearance rates are fast leading to repeated intravitreal injections. Rare complications of intravitreal injections are endophthalmitis, retinal detachment, and vitreous hemorrhage [8–10].

Sustained-release corticosteroid implants have emerged to bridge the problems related to intravitreal injections. These implants reduce the number of injections minimizing the adverse effects of multiple injections, increasing patient comfort and adherence to the treatment while being able to deliver the drug with a lower dose, reducing drug-related adverse events [5].

Currently, three synthetic intravitreal glucocorticoids are used to treat posterior eye segment diseases: triamcinolone (TA), dexamethasone (DEX), and fluocinolone acetonide (FAc). TA is used as an injectable suspension while DEX and FAc require sustained-release delivery systems. While TA and DEX implants have a therapeutic window of 1–6 months, FAc implants can last 3 years [10–12].

For the posterior segment of the eye, three FAc sustained-release devices have been approved by the United States Food and Drug Administration (FDA): Retisert® 0.59 mg (Bausch & Lomb, Rochester, NY), Yutiq® 0.18 mg (EyePoint, Watertown, MA), and Iluvien® 0.19 mg (Alimera Science, Alpharetta, GA).

The 0.19 mg FAc implant has been approved for the treatment of diabetic macular edema and cystoid macular edema related to noninfectious uveitis [10]. However, due to its unique characteristics, ophthalmologists have used this implant to treat other retinal diseases. This review will focus on the off-label use of the 0.19 mg FAc implant.

## 2. 0.19 mg Fluocinolone Acetonide Implant

The 0.19 mg FAc implant is a nonbiodegradable and nonerodible intravitreal implant; it is a light brown colored cylinder with approximately 3.5 mm in length and 0.37 mm in diameter that contains 0.19 mg of FAc. FAc is a synthetic, fluorinated glucocorticoid that is 24 times less soluble than DEX, which allows a prolonged drug release [10, 13]. It is inserted via the pars plana through a 25-gauge injector system as an intravitreal injection, during an in-office procedure, under controlled aseptic conditions, without the need for sutures [14]. It releases 0.25 *μ*g/day (average rate of 0.2 *μ*g/day), up to 36 months [10, 13].

In Europe, it is approved for the treatment of vision impairment associated with chronic DME that is insufficiently responsive to available therapies. In the USA, it is approved for the treatment of DME in patients who have been previously treated with a course of corticosteroids and did not have a clinically significant rise in intraocular pressure (IOP) [15]. More recently, in March 2019, the FAc implant was approved in 17 European countries for the prevention of relapse in recurrent noninfectious uveitis affecting the posterior segment of the eye [10].

The safety and efficacy of the implant were assessed in the FAME (Fluocinolone Acetonide for Diabetic Macular Edema) trials [16], which were parallel-group, prospective, randomized, double-masked, sham-injection controlled, multicenter, phase III clinical trial conducted over a period of 36 months. In this study, two release rates (0.2 and 0.5 *μ*g/day) were compared with sham injections. At 36 months, 28.7% and 27.8% of the patients in the low and high dose, respectively, had a ≥15 letter gain in the Best Corrected Visual Acuity (BCVA) letter score, compared to 18.9% in the sham group. The most common adverse effect was cataract (42.7% low-dose group, 51.7% high-dose group, and 9.7% sham group). Cataract surgery was performed in 80.0% of the patients in the low-dose group, 87.2 in the high-dose group, and 27.3% in the sham group. Another very common adverse effect was the increase of IOP, affecting 37.1% in the low-dose group, 45.5% in the high-dose group, and 11.9% in the sham group.

Also, Campochiaro et al. [17] described the pharmacokinetics and pharmacodynamics in the FAMOUS study. The results showed that the FAc concentration peak occurred on day 7 for most subjects. During the next 6 months, aqueous humor concentrations of FAc decreased. Through months 12 to 36, the concentrations stayed stable. It was also demonstrated that the drug was not detected in the systemic circulation; FAc plasma concentrations were always below the lower limit of quantification of the assay (100 pg/mL) [17, 18].

The 0.19 mg FAc implant is contraindicated in the presence of preexisting glaucoma or active or suspected ocular or periocular infections. In addition, it is contraindicated in patients that have hypersensitivity to the active substance or to any of the excipients of the implant, such as polyvinyl alcohol, polyimide tube, and silicone adhesive [10].

At the moment of this review, simultaneous treatment of both eyes is not recommended until a response to the first implant is known [10].

## 3. Materials and Methods

This systematic review was designed according to the Preferred Reporting Items for Systematic Reviews and Meta-Analysis (PRISMA) criteria [19].

### 3.1. Literature Search Strategy

A literature search on September 20 was conducted in the following electronic databases: Pubmed, Scopus, and Web of Science (from January 1^st^, 2000, to September 20^th^, 2020). The following query was used: (“Fluocinolone Acetonide” OR Iluvien^®^) AND (“eye” OR “ocular” OR “intravitreal).” Furthermore, a hand search of the bibliographies of the included studies and reviews was conducted to identify additional studies.

**Figure 1:**
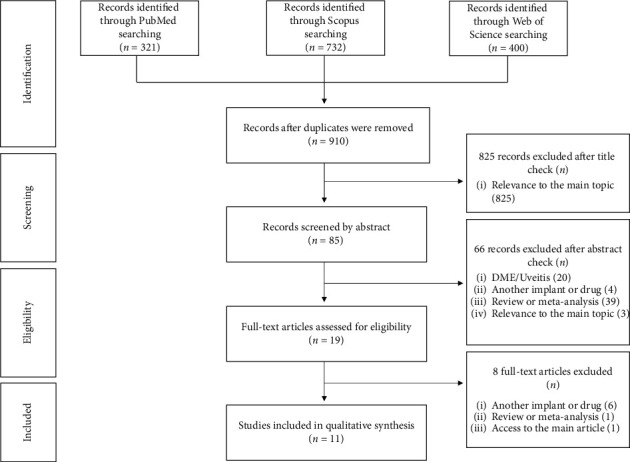
Flowchart, adapted from PRISMA [19], summarizing the selection process for studies concerning off-label FAc implant in ophthalmological diseases.

The search strategy is represented in Figure 1.

### 3.2. Inclusion and Exclusion Criteria

We included studies in which FAc implant was used off-label to treat other ophthalmological diseases besides DME or uveitis.

We excluded, from this review, studies in which FAc implant was used to treat DME or uveitis, studies that used other implants or drugs, reviews or meta-analysis, and other studies that were not relevant to the main topic.

Beyond the temporal limit, no language, age, country, or species restrictions were implemented. The temporal limit was used because the 0.19 mg FAc implant is a recent implant; therefore, there was no need to expand the search to previous years [16].

A total of 11 studies were included in this review based on the inclusion and exclusion criteria mentioned above.

### 3.3. Study Selection

A total of 1453 articles were obtained. Duplicates were removed. Titles of the remaining articles were screened, and those with potential relevance, based on the use of 0.19 mg FAc implant or FAc in other diseases besides DME and uveitis, had their abstracts checked.

### 3.4. Data Extraction

Study details were extracted as follows: disease, author, country and year, type of study, number of subjects, age of the subjects, number of eyes studied, follow-up, and main study conclusions.

## 4. Results

A total of 1453 articles were obtained after a literature search (PubMed: 321, Scopus: 732, and Web of Science: 400). After duplicates were removed, 910 were analyzed. The eligibility was accessed in 19 studies, and a total of 11 were included in this review. Of these 11 studies, 6 are case reports [20–25], 1 is a case series [26], 1 is an abstract [27], 1 is a retrospective study [28], and 2 are randomized control trials [29, 30]. The main characteristics of the included studies are presented in Table 1.

The results are categorized by disease. The diseases included in this review are radiation-induced maculopathy (RM); paraneoplastic visual syndromes (melanoma-related retinopathy (MAR) and cancer-related retinopathy (CAR)); Sjogren's syndrome-related keratopathy; retinal vein occlusion (RVO); cystoid macular edema (CME); diabetic retinal neurodegeneration (DRN); and retinitis pigmentosa (RP).

### 4.1. Radiation-Induced Maculopathy

RM is a common complication after radiotherapy for uveal melanoma and neck and head malignancies. Higher doses of radiation and preexisting diabetes mellitus (DM) are the main risk factors for RM [31]. RM clinical features are similar to those found in diabetic retinopathy. Macular edema is an early and common finding in RM, which causes a decrease in visual acuity or loss of vision [32].

Zimmerman et al. [26] reported the use of FAc implant in 5 patients, 2 males and 3 females (5 eyes: 4 pseudophakic and 1 phakic), aged between 37 and 68, with RM.

All five patients before FAc implant were treated with intravitreal TA injections or DEX implants.

At the end of the follow-up (2–8 months), all five patients had a decrease in foveal thickness. Four of the five patients improved their anatomical retinal structures and BCVA. The only adverse event reported was the increase in IOP in one patient, who required medical therapy. No other adverse event occurred; inclusively, no cataract formation was documented in the phakic eye after 8 months.

The FAc implant, based on these case reports, appears to be a safe and effective treatment for RM when patients are not responsive to the initial treatment. However, as these are case reports, the level of evidence is low; therefore, more studies with longer follow-ups and larger cohorts are needed to validate this data and to determine the efficacy and safety of FAc implant in patients with RM.

### 4.2. Paraneoplastic Visual Syndromes

Paraneoplastic visual syndromes are a heterogeneous group of disorders associated with a systemic malignancy that is not related to the eye [33]. These manifestations can be presented months before the presentation of main cancer [34]. In our literature search, FAc implant was used in cancer-associated retinopathy (CAR) and melanoma-associated retinopathy (MAR), two paraneoplastic syndromes with chorioretinal involvement.

#### 4.2.1. Melanoma-Associated Retinopathy

MAR is a rare autoimmune condition. It is a paraneoplastic syndrome that occurs in patients with cutaneous melanoma, causing progressive visual field loss and night blindness [35].

Karatsai et al. [20] reported a case of a 73-year-old woman followed for 3 years after FAc implant in both eyes.

At the time of treatment, the patient had a BCVA of 20/80 in the right eye, 20/40 in the left eye, and worsening night blindness. One week after the implant, both eyes presented an improvement in visual symptoms, visual field, and BCVA was 20/20. The BCVA remained 20/20 during the 3 years of follow-up. Improvement in retinal function, measured by electroretinography (ERG), was present 1 year after the implant in the right eye and 2 years after the implant in the left eye.

Both eyes developed cataracts 2 years after the implant, requiring cataract surgery. IOP remained normal during the follow-up.

This case report suggests that sustained-release corticosteroid implants, in this case, FAc implant, may be effective in MAR; however, a single case report provides low evidence about the efficacy and safety of FAc implant in patients with MAR. Therefore, more studies are needed to confirm these findings.

#### 4.2.2. Cancer-Associated Retinopathy

CAR is considered the most common intraocular paraneoplastic syndrome. It is caused by antibodies against retinal antigens and it is characterized by painless and progressive visual loss, photosensitivity, ring scotoma, and loss of rod and cone function observed in ERG [34, 36].

Abdulla and Taylor [27], in a retrospective observational clinical study of 6 patients (11 eyes), compared the use of FAc implant (7 eyes) with intravenous immunoglobulin (IVIg) (4 eyes) in CAR, during a follow-up of 6 months.

Six months after the implant, BCVA improved in the FAc group and declined in the IVIg group. IOP dropped in the FAc implant group but increased in the IVIg group. Both groups reported a subjective improvement in vision, particularly in color vision, and an improvement in mean deviation (MD) of the visual field scores. The FAc implant group, compared to the IVIg group, did not require multiple treatments, allowing a better quality of life for these patients.

During the follow-up, the only reported adverse event was cataract surgery in one eye of a patient from the FAc implant group.

FAc implant may have a potential palliative role in patients with CAR. However, only the abstract is available; therefore, it was not peer-reviewed. More studies with larger cohorts, longer follow-ups are needed to validate these results and to determine the efficacy and safety of FAc implant in patients with CAR.

### 4.3. Sjogren's Syndrome-Related Keratopathy

Sjogren's syndrome (SS) is a chronic autoimmune disease characterized by the destruction of exocrine glands, resulting in secretary dysfunction. The typical manifestations are xerostomia and xerophthalmia [35].

Wasielica-Poslednik et al. [21] reported a case of a 52-year-old Caucasian woman with SS secondary to autoimmune hepatitis and primary sclerosing cholangitis which presented to the emergency room with corneal ulcers in both eyes. The left eye had corneal perforation and BCVA was hand motion. The right eye had deep corneal melting and BCVA was 20/100. The patient had a history of filiform keratitis and recurrent erosions for the previous 3 years.

Both eyes underwent penetrating keratoplasty (PKP) and amniotic membrane transplant (AMT). FAc implant was inserted in the left eye, 3 months after her first admission to the emergency room, because of new signs of corneal melting. 2 weeks after the implant, the patient underwent a third PKP and AMT, and a tarsorrhaphy. The right eye served as control. Both eyes equally received topical and systemic therapies.

No additional intervention was performed in the left eye during the 6 months after the third PKP and AMT. On the other hand, the right eye had 2 PKPs, 1 vitrectomy, 5 AMTs, and 3 tarsorrhaphies, due to recurrent keratolysis and perforation.

BCVA in the implanted eye improved from hand motion to 16/100 and no alteration in IOP was documented.

These results suggest that FAc implant may supplement other topical and systemic therapies used in patients with autoimmune corneal diseases; however, this is a single case report; therefore, more studies with longer follow-ups and larger cohorts are needed to obtain a better understanding of the efficacy and safety of FAc implant in autoimmune corneal diseases.

### 4.4. Retinal Vein Occlusion

RVO is one of the main causes of sudden, painless vision loss in adults older than 50 years old. It is related to macular edema and elevated levels of vascular endothelial growth factor (VEGF) and inflammatory mediators. These characteristics make corticosteroids a useful class for their treatment [37].

Coelho et al. [22] reported a case of a 65-year-old man with vision loss in the left eye, with neither risk factors nor ophthalmological history. The patient was diagnosed with central retinal vein occlusion (CRVO) in the same eye a week before and was medicated with oral deflazacort 30 mg and nepafenac 1 mg/ml. His BCVA was 20/100 in the left eye and 20/20 in the right eye. The IOP was normal, and the swinging flashlight test was normal. Optical coherence tomography (OCT) showed peripapillary hemorrhage and papillary edema with exudates. Fluorescein angiography was also performed and demonstrated a delay in arteriovenous transit time in the left eye, retinal hemorrhage, vessel wall staining with perfusion over the peripheral retina, and no visible neovascularization. During a period of 20 months, the patient was treated with 6 DEX implants before treatment with FAc implant. IOP was 16 mmHg, controlled by medication. The patient was pseudophakic at the time of the implant.

The results presented by Alfaqawi et al. [24] showed that from the 1^st^ to the 12^th^ month after the implant, visual acuity improved to 20/25 (before 20/100) and the central subfield foveal thickness decreased from 578 *μ*m to 393 *μ*m. IOP remained stable, controlled by the same medication as when the implant was injected. In the 12^th^ month, it was still possible to observe residual macular edema on OCT.

CRVO macular edema persists for a long time; consequently, a prolonged treatment is needed. FAc implant allows this prolonged treatment without the burden of multiples injections or multiples implants over a short period of time.

The results suggest that FAc implant may be an alternative in patients with CRVO that are not responsive to other treatments. However, this is a single case report, and its level of evidence is low. More studies with longer follow-ups and larger cohorts are needed to validate this data and to determine the efficacy and safety of FAc implant in patients with CRVO.

### 4.5. Cystoid Macular Edema

#### 4.5.1. Persistent Nondiabetic Cystoid Macular Edema after Vitrectomy

Ong et al. [23] reported a case of a 79-year-old Caucasian, a pseudophakic man with CME in both eyes associated with an idiopathic epiretinal membrane (ERM) in the right eye and vitreomacular traction syndrome (VMT) in the left eye. He underwent pars plana vitrectomy with ERM and internal limiting membrane peeling in the right eye, and vitrectomy with intraocular gas tamponade in the left eye. Postoperatively, he had persistent CME in both eyes. In this case, the pathophysiology of postoperative CME was presumed to be inflammatory in nature, caused by the surgery. Therefore, the use of sustained-release corticosteroid implant may have a role in its treatment.

The patient did anti-VEGF injections and intravitreal TA, but CME continued relapsing. Therefore, FAc implant was used first in the left eye and two months later in the right eye. At the time of the implant, BCVA was 20/80 in the left eye and 20/64 in the right eye.

At 13 and 11 months after injection, the left and right eyes, respectively, both eyes demonstrated BCVA improvement, 20/40 (before 20/80) in the left eye and 20/50 (before 20/64) in the right eye. OCT also showed anatomical improvements, with a decrease in central subfield thickness, from 565 *μ*m to 287 *μ*m in the left eye and from 592 *μ*m to 288 *μ*m in the right eye.

No adverse events were reported during the follow-up.

This case shows that the longer duration of FAc implant allows the patient to stay CME free for a longer period.

The results suggest that FAc implant may be an effective and safe therapeutic option for persistent nondiabetic CME after vitrectomy. However, this is a case report, and a generalization of these results has to be made with caution. Therefore, more studies with longer follow-ups and larger cohorts are needed to validate these findings.

#### 4.5.2. Refractory Cystoid Macular Edema after Retinal Detachment Repair

Alfaqawi et al. [24] reported a case of a 65-year-old woman that developed retinal detachment (RD) due to proliferative vitreoretinopathy after right eye pars plana vitrectomy. RD repair was performed. Four months later, the patient developed CME in her right eye and presented a BCVA of 6/36 in the same eye. During a period of 64 months, the patient underwent 3 posterior sub-Tenon's TA injections, 2 intravitreal TA injections, 4 intravitreal DEX implants, and 1 intravitreal anti-VEGF injection. The patient ended up receiving a FAc implant 2 years after the last injection of anti-VEGF, because of refractory DME to the previous treatments.

During the first week, the patient developed mild right eye anterior uveitis. IOP was 27 mmHg in the right eye and 18 mmHg in the left eye. She received IOP lowering medication for 4 weeks. Twenty months after receiving the FAc implant, the patient presented dry fovea on the OCT and BCVA of 6/18 in the right eye.

Inflammation is a probable factor involving CME after retinal detachment repair [38]; therefore, the use of sustained-release corticosteroid implant may have a role in its treatment.

This case report demonstrated that FAc implant allowed a longer period without any additional treatment for CME.

The results suggest that FAc implant may be an effective therapeutic option for refractory CME after RD repair. However, this is a case report and generalizations of these results have to be made with caution. For this reason, more studies with longer follow-ups and larger cohorts are needed to validate these findings.

#### 4.5.3. Severe Iris-Lens Diaphragm Disruption and Recalcitrant Cystoid Macular Edema

Eyes that underwent complicated cataract surgery or posterior capsule rupture have an increased risk for CME, requiring sustainable medical treatment in the long term [38]. They are also at a higher risk to develop complications, such as migration of the implant into the anterior chamber.

Herold et al. [25] reported two patients with disrupted anterior-posterior segment border and persistent CME. Both patients (1 eye each) were treated with a DEX implant, and both patients experienced problems related to the implant or migration of the implant into the anterior chamber.

Herold et al. [25] developed a technique named Fluocinolone Loop-Anchoring Technique (FLAT) with the goal to eliminate the risk of anterior migration of the implant in severely damaged eyes.

After the surgery, both patients experienced subjective discomfort in the suture area due to mechanical disturbance. This discomfort resolved after 4 weeks. There were no problems related to IOP elevation or hypotonia during the first 4 weeks. No migration of the FAc implant was observed in the first 4 weeks.

Both patients showed morphological improvement, with a reduction of central retinal thickness in the spectral domain OCT of >10% during the first 4 weeks after the procedure. BVCA increased one line in one eye and remained stable in the other patient.

This study shows that FLAT may be a solution to improve the treatment in these eyes that are at a higher risk of anterior chamber implant migration. However, a follow-up of only 1 month is not ideal, since the FAc implant lasts up to 36 months. More studies with longer follow-ups and larger cohorts are needed to evaluate the long-term safety and complication rate of this new technique, as well as the efficacy of FAc implant in such eyes.

### 4.6. Diabetic Retinal Neurodegeneration

DRN is a complication of diabetes, and, presently, there are no neuroprotective treatments in patients with diabetes.

Lynch et al. [28] analyzed the area around the fovea of 130 patients, 160 eyes (110 pseudophakic, 38 phakic, 12 not recorded), to calculate the neurodegeneration rate after FAc implant treatment. Two different areas were studied using OCT imaging: zone 1 was located within 1.5 mm of the fovea, and zone 2 was from 1.5 mm to 3.0 mm from the fovea.

Zone 1 showed no statistically significant differences between pre- and post-FAc implant treatment. On the other hand, the rate of thinning in zone 2 was statistically significant, going from 4.0 *μ*m/y before FAc implant to 1.1 *μ*m/y after FAc implant treatment.

This study showed that neuroretinal thinning slowed after treatment with FAc and suggests a potential role of FAc implant in diabetic patients with progressive macular thinning. However, as this was a retrospective study, conclusions have to be made with caution. The authors suggest that a multicenter, prospective, randomized, sham-controlled trial in a population of people with diabetes who have not been diagnosed with DME or diabetic retinopathy would be the ideal study design.

### 4.7. Retinitis Pigmentosa

RP is a group of inherited diseases in which patients present with decreased night vision and loss of peripheral vision because of photoreceptor cell loss.

From the principle that microglial cells are responsible for the neuroinflammation present in many neurodegenerative diseases, Glybina et al. [29, 30], in two RCTs, studied the neuroprotective properties of intravitreal FAc in rats. One study focused on photoreceptor neuroprotection [29] and the other on retinal degeneration [30].

#### 4.7.1. Photoreceptor Neuroprotection

In their first study [29], four groups of Royal College of Surgeons (RCS) rats, 5-week-old, were created: 0.2 *μ*g/d FAc-loaded intravitreal drug-delivery implant (IDDI), 0.5 *μ*g/d FAc-loaded IDDI, inactive IDDI, and nonsurgical control.

The 4-week follow-up time showed no cataract formation, no inflammatory changes, or retinal detachment. IOP showed no statistically significant differences between groups.

ERG amplitudes reduced 80% in the control groups, whereas in the FAc-treated groups no statistically significant attenuation of the ERG amplitudes was observed.

The histologic evaluation demonstrated that, in the FAc-treated groups, the retinal outer nuclear layer (ONL) thickness was greater than that in the control groups, but it was more significant in the 0.2 *μ*g/d group. Retinal inner nuclear layer (INL) thickness was not different between groups.

When counting microglial cells, the FAc-treated groups presented fewer activated cells and fewer microglial cells in the photoreceptor cell layer. These results were greater in the group treated with 0.2 *μ*g/d of FAc.

These results suggest that FAc treatment in patients with RP could help deaccelerate photoreceptor cell death by reducing neuroinflammation.

#### 4.7.2. Retinal Degeneration

In their second study [30], four groups of heterozygote S334ter-4 rat retinal degeneration model (a mutation identical to the human rhodopsin mutation in RP) were created: 0.2 *μ*g/d FAc-loaded intravitreal drug-delivery implant (IDDI), 0.5 *μ*g/d FAc-loaded IDDI, inactive IDDI, and nonsurgical control.

The 8-week follow-up period did not show any signs of infection, inflammation, media opacities, or increase in IOP in any animal.

ERG a-wave amplitudes did not change significantly in the 0.2 *μ*g/d treated group when compared to the other three groups, in which a significant a-wave loss was observed. ERG b-wave amplitude in the FAc-treated eyes was significantly greater than that in the control groups.

Histologic evaluation showed that eyes from the 0.2 *μ*g/d treated group had a significantly thicker ONL than in the other three groups. INL thickness was not different between groups.

Microglial cell reduction was observed in both FAc-treated groups.

The results in this study were consistent with those observed in the RCS rat retinal degeneration model [29].

This study showed that treatment with an FAc implant was associated with the preservation of retinal electrophysiology, morphology, and suppression of retinal microgliosis.

Both studies by Glybina et al. [29, 30] showed that the 0.2 *μ*g/d FAc implant had better results than the higher dose implant.

These two studies suggest that sustained release of FAc may be a future option to treat people with RP by reducing retinal degeneration and by protecting photoreceptors. It would be important to reproduce this study with human subjects, in order to analyze the implant efficacy and safety.

## 5. Discussion

The main purpose of this systematic review was to understand in which other ophthalmological fields FAc implant is being used off-label. With this in mind, a literature search for studies in which FAc implant was being used off-label was performed.

The implant's small size, the release of FAc for 36 months, allowing the patient to stay treatment free for a longer period, and the fact that it is administered by a minimally invasive procedure are all factors of interest to use this implant, especially when patients do not respond to the primary treatment [10].

In this review, a total of 11 studies were included, and the implant was used off-label in a variety of different eye-related diseases. However, the majority of the papers were of low evidence, and the 2 RCTs were performed in nonhuman subjects. Thus, all this data needs to be analyzed with caution, and most of the conclusions from these papers cannot be generalized.

One thing in common with the diseases reviewed is the inflammatory component associated with them. A lot of these diseases already have corticosteroids as part of their therapeutic options; however, the chronicity of these diseases makes the use of some of these corticosteroids a challenge. In most reported cases, the decision to use the FAc implant arose after patients underwent treatment with other corticosteroids that were effective, but their condition ended up relapsing after a short period of time. For example, the DEX implant, which was used in some of the cases reviewed, releases DEX for 3–6 months. Normally, patients need more than one implant for their condition, resulting in more than one procedure needed during treatment [22, 26]. Other therapies used were intravitreal injections with TA [23, 26] or with anti-VEGF [23], which also involve multiple injections. This causes discomfort in the patient and increases the risk of adverse events associated with the injection.

One of the most analyzed outcomes was BCVA. From the results, most patients showed an improvement in their BCVA during follow-up after FAc implant. OCT evaluation also showed anatomical improvements with retinal thickness and macular edema reduction. During follow-up, patients did not need any extra intervention, providing an increased quality of life.

The studies where neuroprotection was studied by Gragoudas et al. [31, 32] concluded that FAc IDDI reduced neuroinflammation mediated by microglial cells. These studies compared two release rates, and 0.2 *μ*g/d was the one with the best results regarding microglia cell reduction and overall neuroinflammation reduction. These results suggest that FAc implant could have a therapeutic role in patients with RP because of photoreceptor cell preservation, as well as in other diseases associated with neuroinflammation.

Lynch et al. [28], in their study, noticed a reduction in neuroretinal thinning rate after FAc implant; consequently, FAc could have a role in patients with DM that are at higher risk of retinal neurodegeneration. However, this was a retrospective study, presenting the limitations inherent to the study design.

The case report presented by Wasielica-Poslednik et al. [21] used FAc implant to treat keratopathy, which is not a disease related to the posterior segment of the eye. This case showed that sustained release of FAc may be used as a supplement to other therapies used to treat keratopathy associated with SS. The eye treated with the implant presented a significant improvement when compared to the control eye. This may open new possibilities for using the FAc implant in inflammatory diseases of the anterior segment.

The most common adverse events related to the FAc implant are cataracts requiring surgery and an increase in IOP [10, 16]. The results corroborate this information. Observing the results of the reviewed papers, we can see that patients with phakic eyes developed cataracts and required surgery. IOP was also measured in most studies, but the elevation of IOP was not as common as the development of cataracts, and when it occurred, it was easily controlled with medication.

However, most of the studies had a short follow-up, and it was not possible to evaluate if the reported effective and safe release of FAc was sustained for 36 months for these different pathologies.

One of the included studies presented a new technique to implant FAc implant. FAc implant normally stays free inside the vitreous cavity, which can result in the migration of the implant into the anterior chamber. Although being uncommon, it has a higher probability of occurring when the patient has a disrupted anterior-posterior segment border after complicated cataract surgery [10, 39]. Herold et al. [25] developed FLAT to treat two patients with disrupted anterior-posterior segment border with persistent CME. The procedure was well tolerated by the patients and just some discomfort in the suture area was documented. No IOP elevation was verified, and no migration of the implant was registered during the 4-week follow-up period. This follow-up is too short to make valid conclusions about the technique and the overall effect of the implant in these patients. It would be important to have these cases studied for a longer period of time to analyze the overall outcome of the implant in their CME. Additionally, a longer follow-up would allow evaluating if the technique is successful, as well as if it is safe.

Overall, these results show that it may be possible for FAc implant to be used in many other eye-related diseases; however, higher-quality studies with well-defined methodologies are needed to corroborate these findings.

## 6. Conclusions

The results presented in this review support the potential use of FAc implant in other ophthalmological diseases besides DME and noninfectious uveitis. The majority of studies included in this review were of low evidence. Nevertheless, these types of studies are important to start a discussion in the community. For these reasons, better methodological studies, such as RCTs with larger cohorts and longer follow-ups, would be important for a greater level of understanding of the efficacy and safety of the potential use of this implant in other ocular diseases.

## Figures and Tables

**Table 1:** Main characteristics of the included studies.

Disease		Author and year	Type of study	Number of subjects	Number of eyes	Follow-up	Conclusions
Radiation-induced maculopathy		Zimmerman et al. [26], Germany	Case series (peer-reviewed)	5 (37–68 years)	5: 4 pseudophakic and 1 phakic	2–8 months	5 patients presented a decrease in foveal thickness. 4 patients improved their retinal structures and visual acuity. Adverse events: 1 patient presented IOP elevation (required medical therapy).
Paraneoplastic syndromes	MAR	Karatsai et al. [20], United Kingdom	Case report (peer-reviewed)	1 (73 years old)	2: 2 phakic	36 months	BCVA left eye: from 20/40 to 20/20. BCVA right eye: from 20/80 to 20/20. ERG with retinal function improvement in both eyes (1 year after in the right eye and 2 years after in the left eye). Adverse events: both eyes developed cataracts 2 years after, requiring cataract surgery.
	CAR	Abdulla and Taylor [27]	Retrospective (not peer-reviewed)	6	11 eyes: (7 treated with FAc implant and 4 treated with IVIg)	6 months	FAc implant outcomes: BCVA improved; MD improved; IOP dropped; color vision improved; did not require multiple treatments or multiple hospital admissions. FAc implant adverse events: One eye from the FAc implant group underwent cataract surgery. IVIg outcomes: BCVA declined; MD improved; IOP increased; color vision improved; did require multiple treatments or hospital admission.
Sjogren's syndrome-related keratopathy		Wasielica-Poslednik et al. [21], Germany	Case report (peer-reviewed)	1 (52 years old)	2 eyes (left eye was treated with FAc implant and the right eye served as a control)	6 months	FAc implant outcomes: BCVA improved from hand motion to 16/100; No more surgical interventions were needed; No alteration in IOP was documented. Control eye: 2 PKPs, 1 vitrectomy, 5 AMTs, and 3 tarsorrhaphies because of recurrent keratolysis and perforations.
Retinal vein occlusion		Coelho et al. [22], Portugal	Case report (peer-reviewed)	1 (65 years old)	1:—1 pseudophakic	12 months	BCVA improved from 20/100 to 20/25. Central foveal thickness decreased from 578 *μ*m to 393 *μ*m. After 12 months, there was still some edema on the OCT. IOP maintained normal, controlled by medication.
Cystoid macular edema	After vitrectomy	Ong et al. [23], USA	Case report (peer-reviewed)	1 (79-year-old)	2:—2 pseudophakic	11–13 months	BCVA left eye: from 20/80 to 20/40. BCVA right eye: from 20/64 to 20/50. Both with anatomical improvement on OCT. IOP remained normal.
	Retinal detachment repair	Alfaqawi et al. [24], United Kingdom	Case report (peer-reviewed)	1 (65 year-old)	1	20 months	BCVA improved from 6/36 to 6/18. Anatomically, presented dry fovea. Adverse events: Anterior uveitis during the first week after surgery and elevated IOP, which was controlled with medication for 4 weeks.
	Disrupted anterior-posterior segment border (FLAT)	Herold et al. [25], Germany	Case report (peer-reviewed)	2	2	1 month	BCVA improved one line in one patient and stayed the same in the other >10% reduction of central retinal thickness on OCT in both patients. Adverse events: Subjective discomfort in the suture area that resolved during the first 4 weeks.
Diabetic retinal neurodegeneration		Lynch et al. [28], USA	Retrospective (peer-reviewed)	130 (mean age 69.6 years)	160:—110 pseudophakic - 38 phakic—12 not recorded	Mean follow-up post FAc implant: 13.2 months	Region 1: The rate of inner retinal loss was not statistically significant. Region 2: The rate of inner retinal loss was statistically significant. The rate of thinning went from 4.0 *μ*m/y to 1.1 *μ*m/y after FAc implant treatment.
Retinitis pigmentosa	Photoreceptor neuroprotection	Glybina et al. [29], USA	RCT (peer-reviewed)	32 RCS rats	64	4 weeks	ERG: ERG a- and b- wave amplitudes were preserved in all animals receiving FAc implants. There was an 80% reduction in the control groups. This preservation in the FAc implant groups was statistically significant compared to control groups. Retinal histology: ONL thickness was greater than in the control groups. INL thickness was not different between groups. Microglial cell count: FAc significantly reduced the overall number of microglial cells in the photoreceptor cell layer, when compared to the control groups. The number of activated microglial cells was also reduced. Adverse events: No cataract formation, no inflammatory changes, or retinal detachment. IOP showed no statistically significant differences between groups. Overall conclusion: The 0.2 *μ*m/d FAc implant was reproducibly more effective than the higher dose implant.
	Retinal degeneration	Glybina et al. [30], USA	RCT (peer-reviewed)	32 S334ter-4 rats	64	8 weeks	ERG: ERG a-wave amplitudes did not change significantly in the 0.2 *μ*m/d group. ERG b-wave amplitudes were greater in the FAc groups than in the control groups. These observations indicate that steroid treatment was associated with the preservation of rod-driven electrical activity during phototransduction. Retinal histology: The 0.2 *μ*m/d FAc implant group presented a thicker ONL. INL thickness was not significantly different between groups. Microglial cell count: Microglial cell count was significantly lower in both FAc implant groups when compared to the control groups. Adverse events: No signs of infection, inflammation, media opacities, or IOP were observed during the 8-week postoperative period in any animal. Overall conclusion: The 0.2 *μ*m/d FAc implant was reproducibly more effective than the higher dose implant.

## Data Availability

No new research data were used to support this study.

## References

[1] Sarao V., Veritti D., Boscia F., Lanzetta P. (2014). Intravitreal steroids for the treatment of retinal diseases. *Scientific World Journal*.

[2] Felinski E. A., Cox A. E., Phillips B. E., Antonetti D. A. (2008). Glucocorticoids induce transactivation of tight junction genes occludin and claudin-5 in retinal endothelial cells via a novel cis-element. *Experimental Eye Research*.

[3] Congdon N., O’Colmain B, Klaver C. C. (2004). Causes and prevalence of visual impairment among adults in the United States. *Archives of Ophthalmology*.

[4] Gordois A., Cutler H., Pezzullo L. (2012). An estimation of the worldwide economic and health burden of visual impairment. *Global Public Health*.

[5] Neumann R., Barequet D. (2019). The gap between the need for novel retinal drug delivery methods, technologies in R & D phase, and approved ocular drug delivery technologies. *Drug Discovery Today*.

[6] Hughes P., Olejnik O., Changlin J., Wilson C. (2005). Topical and systemic drug delivery to the posterior segments. *Advanced Drug Delivery Reviews*.

[7] Kapugi M., Cunningham K. (2019). Corticosteroids. *Orthopaedic Nursing*.

[8] Lee S. S., Hughes P. M., Robinson M. R. (2009). Recent advances in drug delivery systems for treating ocular complications of systemic diseases. *Current Opinion in Ophthalmology*.

[9] Kurz D., Ciulla T. (2002). Novel approaches for retinal drug delivery. *Ophthalmology Clinics of North America*.

[10] Alimera Sciences Limited (2019). *ILUVIEN 190 Micrograms Intravitreal Implant in Applicator*.

[11] Yang Y., Bailey C., Loewenstein A., Massin P. (2015). Intravitreal corticosteroids in diabetic macular edema. *Retina*.

[12] Whitcup S. M., Cidlowski J. A., Csaky K. G., Ambati J. (2018). Pharmacology of corticosteroids for diabetic macular edema. *Investigative Opthalmology & Visual Science*.

[13] Jaffe G. J., Yang C. H., Guo H., Denny J. P., Lima C., Ashton P. (2000). Safety and pharmacokinetics of an intraocular fluocinolone acetonide sustained delivery device. *Investigative Ophthalmology & Visual Science*.

[14] Kane F. E., Burdan J., Cutino A., Green K. E. (2008). Iluvien: a new sustained delivery technology for posterior eye disease. *Expert Opinion on Drug Delivery*.

[15] Syed Y. Y. (2017). Fluocinolone acetonide intravitreal implant 0.19 mg (iluvien): a review in diabetic macular edema. *Drugs*.

[16] Campochiaro P. A., Brown D. M., Pearson A. (2012). Sustained delivery fluocinolone acetonide vitreous inserts provide benefit for at least 3 years in patients with diabetic macular edema. *Ophthalmology*.

[17] Campochiaro P. A., Nguyen Q. D., Hafiz G. (2013). Aqueous levels of fluocinolone acetonide after administration of fluocinolone acetonide inserts or fluocinolone acetonide implants. *Ophthalmology*.

[18] Campochiaro P. A., Hafiz G., Shah S. M. (2010). Sustained ocular delivery of fluocinolone acetonide by an intravitreal insert. *Ophthalmology*.

[19] Liberati A., Altman D. G., Tetzlaff J. (2009). The PRISMA statement for reporting systematic reviews and meta-analyses of studies that evaluate health care interventions: explanation and elaboration. *Annals of Internal Medicine*.

[20] Karatsai E., Robson A. G., Taylor S. R. J. (2019). Outcomes associated with sustained-release intraocular fluocinolone implants in a case of melanoma-associated retinopathy treated without systemic immunosuppression. *JAMA Ophthalmology*.

[21] Wasielica-Poslednik J., Pfeiffer N., Gericke A. (2019). Fluocinolone acetonide intravitreal implant as a therapeutic option for severe sjögren’s syndrome-related keratopathy: a case report. *Journal of Medical Case Reports*.

[22] Coelho J., Pessoa B., Meireles A. (2019). Long-term management of non-ischemic central retinal vein occlusion with fluocinolone acetonide intravitreal implant 190 *μ*g (ILUVIEN®). *Therapeutic Advances in Ophthalmology*.

[23] Ong S. S., Walter S. D., Chen X., Thomas A. S., Finn A. P., Fekrat S. (2018). Bilateral intravitreal 0.19 mg fluocinolone acetonide implant for persistent nondiabetic cystoid macular edema after vitrectomy. *Retinal Cases and Brief Reports*.

[24] Alfaqawi F., Sarmad A., Ayesh K., Mitra A., Sharma A. (2018). Intravitreal fluocinolone acetonide (ILUVIEN) implant for the treatment of refractory cystoid macular oedema after retinal detachment repair. *Türk Oftalmoloji Dergisi*.

[25] Herold T. R., Liegl R., Koenig S., Almarzooqi A., Priglinger S. G., Wolf A. (2020). Scleral fixation of the fluocinolone acetonide implant in eyes with severe iris-lens diaphragm disruption and recalcitrant CME: the fluocinolone-loop-anchoring technique (FLAT). *Ophthalmology and Therapy*.

[26] Zimmermann L., Kneifel C., Grajewski L., Ciernik I. F., Krause L. (2020). Treatment of radiation-induced maculopathy with fluocinolone acetonide. *Graefe’s Archive for Clinical and Experimental Ophthalmology*.

[27] Abdulla D., Taylor S. R. (2020). Fluocinolone acetonide intravitreal implant 0.19 mg (ILUVIEN®) vs Intravenous immunoglobulin (IvIg) in cancer associated retinopathy. *Investigative Ophthalmology & Visual Science*.

[28] Lynch S. K., Lee K., Chen Z. (2019). Intravitreal fluocinolone acetonide may decelerate diabetic retinal neurodegeneration. *Investigative Opthalmology & Visual Science*.

[29] Glybina I. V., Kennedy A., Ashton P., Abrams G. W., Iezzi R. (2009). Photoreceptor neuroprotection in RCS rats via low-dose intravitreal sustained-delivery of fluocinolone acetonide. *Investigative Opthalmology & Visual Science*.

[30] Glybina I. V., Kennedy A., Ashton P., Abrams G. W., Iezzi R. (2010). Intravitreous delivery of the corticosteroid fluocinolone acetonide attenuates retinal degeneration in S334ter-4 rats. *Investigative Opthalmology & Visual Science*.

[31] Gragoudas E. S., Li W., Lane A. M., Munzenrider J., Egan K. M. (1999). Risk factors for radiation maculopathy and papillopathy after intraocular irradiation. *Ophthalmology*.

[32] Horgan N., Shields C. L., Mashayekhi A., Teixeira L. F., Materin M. A., Shields J. A. (2008). Early macular morphological changes following plaque radiotherapy for uveal melanoma. *Retina*.

[33] Bussat A., Langner-Lemercier S., Salmon A., Mouriaux F. (2018). Paraneoplastic syndromes in ophthalmology. *Journal Français d’Ophtalmologie*.

[34] Rahimy E., Sarraf D. (2013). Paraneoplastic and non-paraneoplastic retinopathy and optic neuropathy: evaluation and management. *Survey of Ophthalmology*.

[35] Vivino F. B., Minerva P., Huang C. H., Orlin S. E. (2001). Corneal melt as the initial presentation of primary sjögren’s syndrome. *The Journal of Rheumatology*.

[36] Naramala S., Ahmad J., Adapa S., Gavini F., Konala V. M. (2019). Case series of cancer-associated retinopathy (CAR). *Cureus*.

[37] Blair K., Czyz C. N. (2020). *Central Retinal Vein Occlusion*.

[38] Flach A. J. (1998). The incidence, pathogenesis and treatment of cystoid macular edema following cataract surgery. *Transactions of the American Ophthalmological Society*.

[39] Tabandeh H., Rezaei K. (2020). Scleral fixation of fluocinolone acetonide implant. *American Journal of Ophthalmology Case Reports*.

